# Semantic control across Alzheimer’s disease phenotypes: cognitive mechanisms and neural correlates

**DOI:** 10.3389/fnagi.2026.1821431

**Published:** 2026-08-12

**Authors:** Pilar Armas, Alejandra O. Morcillo-Nieto, Sara E. Zsadanyi, Sonia Karin Marques-Kiderle, Júlia Filella-Mercè, Francesco Ciongoli, Isabel Sala, M. Belen Sanchez-Saudinos, Sara Rubio-Guerra, Nuole Zhu, Isabel Barroeta, Maria Carmona-Iragui, Ignacio Illán-Gala, Daniel Alcolea, Alberto Lleo, Juan Fortea, Stephanie M. Grasso, Marco Calabria, Javier Hernando, Elizabeth Jefferies, Matthew A. Lambon Ralph, Richard J. Binney, Alexandre Bejanin, Miguel A. Santos-Santos

**Affiliations:** 1 Sant Pau Memory Unit, IR SANT PAU, Hospital de la Santa Creu i Sant Pau, Barcelona, Spain; 2 Universitat Politècnica de Catalunya (UPC), Barcelona, Spain; 3 Centre of Biomedical Investigation Network for Neurodegenerative Diseases (CIBERNED), Madrid, Spain; 4 Institut de Neurociències, Universitat Autònoma de Barcelona, Bellaterra, Spain; 5 Faculty of Health Sciences, Universitat Oberta de Catalunya, Barcelona, Spain; 6 Barcelona Down Medical Center, Fundació Catalana Síndrome de Down, Barcelona, Spain; 7 Department of Speech, Language, and Hearing Sciences, The University of Texas at Austin, Austin, TX, United States; 8 Department of Psychology, University of York, York, United Kingdom; 9 Medical Research Council (MRC) Cognition and Brain Sciences Unit, University of Cambridge, Cambridge, United Kingdom; 10 Department of Psychology, Bangor University, Gwynedd, United Kingdom

**Keywords:** Alzheimer’s disease, controlled retrieval, logopenic variant primary progressive aphasia, neuroimaging, selection mechanism, semantic cognition, semantic control

## Abstract

**Introduction:**

Semantic cognition, our ability to understand meaning, relies on two distinct but interacting systems: one involved in knowledge representation and another in control processes. This control system is thought to comprise at least two mechanisms: controlled retrieval, which facilitates access to information relevant to a specific goal or context, and selection, which is engaged to resolve competition among multiple simultaneously active semantic representations. The present study aimed at understanding if, and how, controlled semantic cognition is impaired in Alzheimer’s disease (AD), and the neuroanatomical correlates of these deficits. We supposed that in AD, semantic deficits could reflect damage to the retrieval and/or the selection mechanism and could differ according to the clinical phenotype of AD.

**Methods:**

Our sample included the typical amnestic (ADtyp, *N* = 11) clinical presentation as well as the atypical language-dominant variant (logopenic variant primary progressive aphasia, lvPPA, *N* = 17). 16 healthy controls also took part. We examined object use knowledge in a task that manipulated the levels of both controlled-retrieval and selection semantic control mechanisms.

**Results:**

Increased semantic control demands worsened performance in all groups including controls. Both patient groups were impaired in controlled retrieval, but not selection, compared to controls. Furthermore, lvPPA scored consistently lower than controls and ADtyp in all conditions of the experimental task, even the condition with the lowest semantic control demands, pointing to broader semantic difficulties beyond controlled-retrieval and selection… Exploratory analyses suggested the two mechanisms were linked to partially distinct predominant cognitive associations, controlled-retrieval with tests of semantic-knowledge and selection with executive function. Neuroimaging showed partially overlapping but distinct correlates for the two mechanisms, potentially reflecting differing reliance on semantic versus domain-general control brain networks.

**Conclusion:**

Our results refine current models of semantic dysfunction in AD, highlighting involvement of both domain-general and more specialized control mechanisms, with potential implications for differential diagnosis, symptom management, and rehabilitation planning.

## 1 Introduction

Semantic cognition is the processes involved in acquiring and using conceptual knowledge, the meaning of words, objects and people gleaned from experience, which guides our understanding of the world (Tulving, 1972; Collins and Loftus, 1975; Smith, 1978). Impairments of semantic cognition arise from a variety of brain injuries and diseases, but they vary qualitatively reflecting the involvement of different neuroanatomy and cognitive mechanisms. These variations have influenced accounts of how semantic knowledge is organized and processed (e.g., Martin, 2016; Patterson et al., 2007, for a review). One prominent theory, the controlled semantic cognition (CSC) framework (Ralph et al., 2017), is primarily based on the distinct impairments observed in patients with semantic dementia (SD) and semantic aphasia (SA) (Corbett et al., 2015; Jefferies and Lambon Ralph, 2006). SD, or semantic variant primary progressive aphasia (svPPA), is a neurodegenerative condition characterized by gradual and relatively selective multimodal deterioration of core semantic representations accompanied by progressive atrophy of the bilateral anterior temporal lobes (Gorno-Tempini et al., 2011; Rogers et al., 2004). In contrast, SA typically occurs in patients with brain damage from stroke affecting the left inferior frontal and posterior temporal parietal regions and is characterized by impaired regulation of semantic representations manifesting as verbal and non-verbal (Corbett et al., 2009; Noonan et al., 2010; Corbett et al., 2011) semantic impairment typified by a set of features including marked sensitivity to task demands, strong cueing and context effects, inconsistent error patterns, and absence of frequency effects among others (Ralph et al., 2017; Jefferies and Lambon Ralph, 2006). Accordingly, the CSC framework holds that semantic cognition is underpinned by two distinct but interacting systems: one involved in knowledge representation and another in control processes. The semantic control system selectively accesses and manipulates semantic representations based on task demands.

Semantic control is thought to include at least two mechanisms that may operate partially independently (Jefferies and Wang, 2021). The first is a controlled retrieval mechanism, which involves accessing knowledge relevant to the current goal or context, especially when automatic retrieval is insufficient. This mechanism has been shown to correlate with tests of the size of the semantic store of knowledge (lexical decision and synonym tests) (Hoffman, 2018; Wu et al., 2024) and activation of left anterior ventral inferior prefrontal and posterior middle temporal cortex (Thompson-Schill et al., 1997; Badre et al., 2005; Whitney et al., 2012). The second process is a selection mechanism, which is engaged to resolve competition between multiple simultaneously active semantic representations. Performance on tasks that tap this ability correlates with performance on executive function and non-semantic control, is selectively impaired in old age, and is associated to activation of the posterior portion of the left inferior prefrontal cortex (Wu and Hoffman, 2022). These features suggest this mechanism engages the domain-general cognitive control network that responds to a multitude of cognitive demands (i.e., the multiple demands network (Duncan, 2010; Camilleri et al., 2018).

Various tests have been used to study semantic control. Controlled retrieval is often probed with semantic association tasks that vary association strength. Trials that require a weak or non-dominant link, such as matching scissors with screwdriver, are harder than trials with a strong link, such as fork with knife, because automatic processing does not activate the target and a goal-directed search is needed. Semantic selection is typically examined with feature-matching tasks that ask participants to match items by a specific feature, such as color or size, while ignoring a strong but irrelevant relation. For example, deciding that salt matches cloud on color while ignoring pepper as a strong but irrelevant associate. In these paradigms, larger accuracy gaps between high versus low control conditions index poorer semantic control.

Studies regarding the nature of semantic impairment in AD have reached mixed conclusions (Aronoff et al., 2006) over the past few decades. According to one view, AD damages semantic knowledge representations (Chertkow and Bub, 1990; Hodges et al., 1992; Rohrer et al., 1999; Giffard et al., 2001), whereas other studies provide evidence of semantic control impairment (Bayles et al., 1991; Nebes and Halligan, 1995; Daum et al., 1996; Bayles et al., 1999). More recently, Corbett et al demonstrated that the nature of semantic impairment in AD is not stable, but rather subject to qualitative changes throughout the course of the disease (Corbett et al., 2012, 2015). Across two studies, they showed, in both the verbal and non-verbal domains, that the semantic impairment exhibited by mild AD patients most closely resembled the pattern observed in SA, indicative of semantic control impairment. In contrast, severe AD patients presented characteristics indicative of degraded semantic representations akin to SD. The status of semantic cognition in atypical variants of AD is less understood, and it may well differ given their different topographies of atrophy patterns. Logopenic variant primary progressive aphasia (lvPPA) is of particular interest given their predominant atrophy in the posterior temporal-inferior parietal region (Gorno-Tempini et al., 2011) overlaps with one of the main areas damaged in SA, raising the possibility that lvPPA also presents one or many features of semantic control impairment. In line with their main presenting symptoms of anomia and impaired sentence repetition (Gorno-Tempini et al., 2004), lvPPA is principally associated with an impairment in phonological processing rather than semantic cognition (Gorno-Tempini et al., 2008; but see Henderson et al., 2025a). However, there is some evidence of impairment at the phonological, lexical, and semantic levels of language processing (Migliaccio et al., 2016; Leyton et al., 2017; Roncero et al., 2020; Santi et al., 2024), as well as reports of a significant proportion of patients with PPA that present a mixed logopenic/semantic variant profile (Sajjadi et al., 2014; Louwersheimer et al., 2016; Mazzeo et al., 2024; Watanabe et al., 2024). Of particular relevance for this study, Henderson et al. (2025b) provide initial evidence of semantic control deficits in lvPPA that manifest across both verbal and non-verbal domains and increase with disease severity. However, significant questions remain regarding the nature of these impairments in different AD syndromes as well as their neural correlates. In particular, the status of different aspects of semantic access impairment (controlled retrieval, selection) have yet to be established. Furthermore, the neural correlates of these mechanisms also warrant further investigation as lvPPA typically present atrophy in the posterior temporal/inferior parietal region but not in the left inferior frontal gyrus (IFG), both key regions in the semantic control network.

We therefore asked the following questions: (i) are individuals diagnosed with typical memory predominant AD and lvPPA impaired in controlled retrieval, semantic selection, or both, and (ii) what are the cognitive and neural correlates of the mechanisms of semantic impairment in AD? Besides refining current models of semantic dysfunction in AD, if typical and atypical AD show differentiable profiles of semantic impairment, our results could improve differential diagnosis as well as symptom tracking and management.

The present study investigated semantic control in different phenotypes of AD, via behavioral and neuroimaging data. We recruited cerebrospinal fluid (CSF) biomarker confirmed AD patients with either the typical memory (ADtyp) or atypical language (lvPPA) presentation, and used an adapted version of the *canonicity and distraction in object selection* test from Corbett et al. (2011). This task contains experimental manipulations that allow evaluation of both controlled retrieval (canonical use manipulation) and selection (distraction strength or relatedness manipulation) in a semantic association task. Previous literature shows contrasting findings, Corbett et al., 2015 found impairment in both mechanisms in ADtyp, whereas Henderson et al. (2025b) report relative preservation in both mechanisms in ADtyp compared to lvPPA, the present study aims to resolve. Regarding the cognitive and imaging correlates, we expected that controlled retrieval would be preferentially associated with executive function and semantic knowledge tests and atrophy in key areas of the semantic control network, such as the IFG and posterior middle temporal gyrus (pMTG), whereas semantic selection would correlate primarily with executive function tests and atrophy in frontal areas included in both the semantic and domain-general cognitive control systems, such as the IFG and middle frontal gyrus (MFG).

## 2 Materials and methods

### 2.1 Participants

We included 44 participants from the Sant Pau Initiative on Neurodegeneration (SPIN cohort) (Alcolea et al., 2019) evaluated at the Sant Pau Memory Unit (Barcelona, Spain) between May 2021 and May 2023. Patients underwent standardized neuropsychological, neurological, neuroimaging (magnetic resonance imaging, MRI), and CSF biomarker evaluation in accordance with the Memory Unit’s usual procedures (Alcolea et al., 2019).

Eleven participants received a diagnosis of ADtyp characterized by an amnestic clinical presentation (Dubois et al., 2014) and seventeen received a diagnosis of lvPPA, presenting predominant impairments in word retrieval and sentence repetition (Gorno-Tempini et al., 2011; Grossman, 2010). All patients underwent lumbar puncture to assess core AD biomarkers (Aβ42/40, total Tau, and pTau181), and all presented with a CSF profile consistent with AD pathology (A+T+). The control group comprised 16 cognitively healthy individuals from the SPIN healthy aging cohort, with no history of neurological or psychiatric disorders. All but one participant were native speakers of Spanish or Catalan, or bilingual in both languages; one control participant was a native Dutch speaker with high proficiency in Spanish. All participants gave written consent, and the ethics committee of Hospital Sant Pau approved all procedures included in this study, in accordance with the Declaration of Helsinki.

#### 2.1.1 Neuropsychological evaluation

For a detailed description of the neuropsychology battery administered in SPIN (see Alcolea et al., 2019). Briefly, the Mini Mental State Examination (MMSE, Blesa et al., 2001) is used to assess global cognitive status. Semantic memory and language are measured using the 60-item Boston Naming Test (BNT), the Order Comprehension test from the Consortium to Establish a Registry for Alzheimer’s Disease (CERAD) battery, and semantic fluency (1 min, animals). Executive functions are evaluated with the Digits Forward and Backward from the Wechsler Memory Scale, phonemic fluency (1 min, “p”), Trail-Making A and B (TMTA/B). Visuospatial, visuoperception, and visuoconstructive abilities are assessed with the Number Location subtest from the Visual Object Spatial Perception (VOSP) battery, the superposition of figures from the Poppelreuter test for visual agnosia, and the Clock-Drawing (command) test and CERAD figures copy test, respectively. Visual memory is evaluated using the CERAD figures recall. Finally, verbal memory is assessed using the Free and Cued Selective Reminding Test (FCSRT). Study participants received additional language tests outside of the usual SPIN protocol that included a short and long sentence repetition test and a synonym test (Noonan et al., 2010), which required selection of a target word among two distractors. Due to standard clinical constraints (patient fatigue, variable testing protocol lengths, and retrospective data availability) not all participants completed the full battery of neuropsychological assessments. Consequently, the sample size (N) varies across specific correlation analyses.

### 2.2 Experiment—canonicity and distraction in object selection

We used the *canonicity and distraction in object selection* test from Corbett et al. (2011), which examines object use knowledge across varying levels of semantic control demands. The test was translated into Spanish, and nine culturally irrelevant items were removed.

#### 2.2.1 Experiment design

**FIGURE 1:**
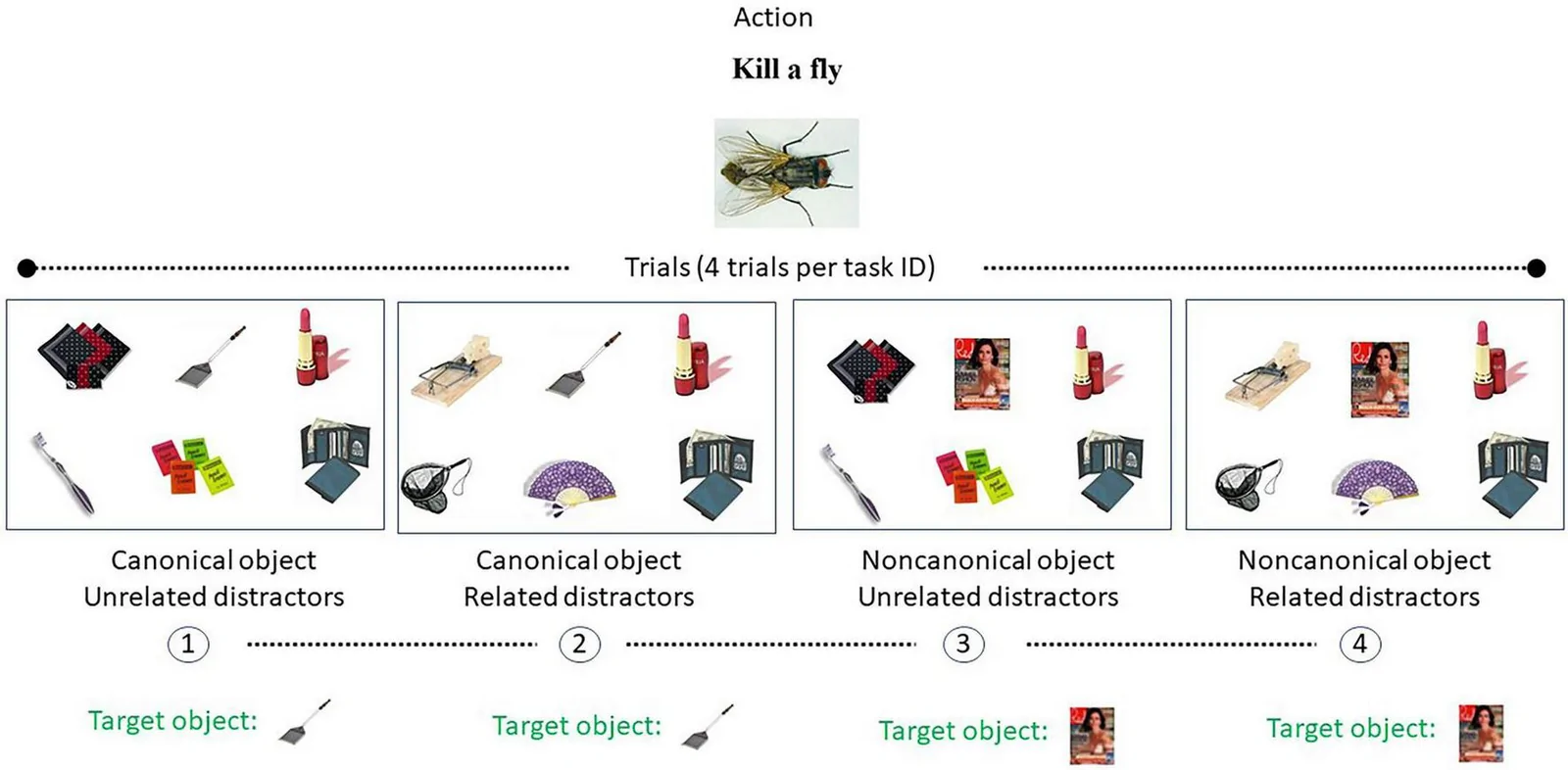
Experimental task (Corbett et al., 2011). The executive demands of controlled retrieval are increased when the canonical object was not present, but a plausible, non-canonical alternative constituted the correct response (e.g., “kill a fly” with a (rolled-up) magazine instead of a fly swatter) and the demands of selection were increased by including related vs. unrelated distractors therefore increasing the difficulty of rejecting them.

Before beginning the experiment, participants completed a practice trial session to ensure they understood the task. Participants used a dual-screen setup, with separate displays for instructions and task stimuli (see Figure 1). On one screen, participants were visually and auditorily presented with a phrase that described an everyday task as well as the target of this task (e.g., “kill a fly” and the picture of a fly). On another screen, the participants saw six color pictures of objects (two rows, three pictures per row) and had to choose the most appropriate object for completing the task by pressing a button. The experiment manipulates the canonicity of the target (the correct choice or target was either a canonical object to complete the task, e.g., “fly swat,” or a non-canonical one, e.g., “a rolled-up magazine”) and the relatedness of the distractors (the distractor objects were either semantically related or unrelated to the correct choice). A total of 28 different everyday actions were assessed under four conditions with a 2 × 2 design: (1) target canonical object with unrelated distractors (C-U), (2) target canonical object with semantically related distractors (C-R), (3) target non-canonical object with unrelated distractors (N-U), and (4) target non-canonical object with semantically related distractors (N-R, see Figure 1). In the conditions with related distractors, three of the five distractors were semantically related to the target, and the remaining two distractors were unrelated objects. In the unrelated conditions, no distractor was semantically linked to the target. The distinction between related and unrelated distractors was marked; while the related distractors came from the same semantic category as the target canonical object and served closely related functions, the unrelated distractors had no functional or categorical relationship with the target. The distractors were identical across the canonical/non-canonical conditions so that any difference across these conditions could only be attributed to changes in the target rather than the distractors. All 28 actions were assessed in each of the 4 conditions, making a total of 112 (4*28) trials. The four conditions were interleaved in a pseudorandom order.

The task developed by Corbett et al. (2011) is specifically structured to isolate and manipulate two distinct mechanisms of semantic control: controlled retrieval and selection. While a patient’s underlying semantic representations are inherently engaged during the task, the systematic manipulation of control demands (contrasting canonical versus non-canonical object uses, and related versus unrelated distractors) is engineered to ensure that the requirements for semantic control can be isolated from basic representational integrity. As shown in Figure 1, the images in the Noncanonical-Unrelated condition (#3) are identical to the Canonical-Unrelated condition (#1) except for the image of the Noncanonical (magazine, which can be rolled-up and swung at a fly to complete the Action “kill a fly”) vs. Canonical (fly-swatter) target object. Following a similar logic, the Canonical-Related condition (#2) differs from the Canonical-Unrelated condition (#1) only in the images of the two distractor objects which change to semantically related distractors (i.e., mouse trap and fishing net).

#### 2.2.2 Outcome measures

Three main scores can be derived from the task. We computed participants’ accuracy for each of the four distinct conditions, similar to Corbett et al., 2015. Additionally, we derived two metrics reflecting different aspects of semantic control: the Canonicity score that measures the impact of the canonical manipulation requiring participants to search for an alternative non-canonical object consistent with increasing demands of controlled retrieval; and the Relatedness score that measures the impact of the type of distractors (related vs. not related) therefore increasing demands of semantic selection processes. The Canonicity and Relatedness scores were calculated by measuring the accuracy difference between trials completed under canonical minus noncanonical and unrelated minus related conditions, respectively, for each action. Therefore, higher Canonicity and Relatedness scores are expected to indicate greater impairment in semantic control, controlled retrieval and semantic selection respectively, capacity.

Semantic representation impairment with preservation of semantic control would result in low overall accuracy scores in all four conditions (critically, a low score in the Canonical-Unrelated condition which requires the least control) and near 0 Canonicity and Relatedness scores. Semantic representation preservation with control impairment would result in the opposite pattern (preserved scores in the Canonical-Related and impaired scores in the other conditions; high Canonicity and Relatedness scores). Impairment in both representation and control would result in low overall accuracy scores across all four conditions and high Canonicity and Relatedness scores.

### 2.3 Neuroimaging acquisition and pre-processing

A subset of 41 participants (16 controls, 11 ADtyp, and 14 lvPPA patients) underwent high-resolution T1-weighted MRI scans using a 3T Siemens Prisma or Philips Achieva and a voxel size of ∼1 × 1 × 1 mm. All T1 images were processed using CAT12 within SPM12 on MATLAB R2019b. Gray matter (GM) segment images were normalized, modulated, and smoothed with a 10-mm Gaussian kernel.

### 2.4 Statistical analysis

#### 2.4.1 Behavioral data analyses

Statistical analyses for the behavioral data were carried out with R software version Rv4.3.0.^1^

##### 2.4.1.1 Demographic and neuropsychological data

Demographic and neuropsychological data were compared across the three diagnostic groups. Continuous variables were analyzed using the non-parametric Kruskal-Wallis test, followed by *post-hoc* pairwise comparisons using Wilcoxon rank-sum tests. Categorical variables (sex) were evaluated using the Chi-squared test, with Fisher’s exact tests applied for subsequent *post-hoc* pairwise comparisons.

##### 2.4.1.2 Experimental task data

*Quality control*: To identify potentially problematic trials, we examined trials with low accuracy in the control group, specifically focusing on those where (i) the overall accuracy for a specific trial was below 50%; or (ii) an incorrect answer was consistently selected over the correct one, meaning the most frequently chosen object was not the target object. Statistical analyses were conducted both including and excluding these trials. Since the exclusion of these trials did not affect the overall results, we chose to report the results that take all trials into account.

*Modeling of task performance:* Global and per-condition accuracy as well as Canonicity and Relatedness scores are reported as medians and interquartile range for all three diagnostic groups. Logistic mixed effects modeling was implemented using the lme4 package to evaluate the likelihood of a correct response at the individual trial level as a function of diagnosis group (three levels: control, ADtyp, lvPPA), the canonicity of the target item (two levels: canonical, noncanonical) and the relatedness of the distractors (two levels: related, unrelated). Participants, actions, and the position of the target on the screen were introduced as random intercepts. Age, gender and education years were included as covariates to control for demographic variations. To investigate potential group differences, we also incorporated the interactions diagnosis × canonicity and diagnosis × relatedness. Finally, to decide whether to include the interaction term canonicity × relatedness, we performed a nested model comparison (see supplementary Table 1) and evaluated various model comparison metrics: likelihood ratio tests (glmer f(x), lme4 package), AICc weights, and marginal/conditional R^2^ (compare_performance f(x), performance package). The results showed that the interaction canonicity x relatedness significantly improved model fit and therefore the final evaluated model was: correct response ∼ Relatedness × DX + Canonicity × DX + Relatedness × Canonicity + Age + Sex + Education years + (1| participant) + (1| Task ID) + (1| correct position).

*Comparison of accuracy across diagnostic groups:* To evaluate for significant differences in accuracy across diagnostic groups, we examined the optimal trial-level accuracy model and performed post-hoc pairwise comparisons with estimated marginal means (*emmeans* package, Lenth, 2023) comparing global accuracy and accuracy for each of the four conditions across the three diagnostic groups. *P*-values were adjusted using Tukey’s Honestly Significant Difference (HSD) method.

*Evaluation of the effects of the canonicity and relatedness experimental manipulations*: Four different analyses were conducted to evaluate the effects of Canonicity and Relatedness and whether these effects differed depending on diagnosis: (1) We evaluated how accuracy varied across each experimental condition within each diagnostic group by evaluating the trial-level accuracy model and performing *post-hoc* within-group pairwise comparisons across all four experimental conditions. (2) We evaluated the diagnosis x canonicity and diagnosis × relatedness interaction terms in the optimal trial-level accuracy model, and (3) we performed condition-specific *post-hoc* pairwise comparisons across its levels. Method (4) employed the Canonicity and Relatedness scores described above instead of the trial-level accuracy model. We compared these scores across diagnostic groups via multiple linear regression models evaluating the main effect of diagnosis on each score while controlling for age, sex, and years of education. Post-hoc pairwise comparisons between the three diagnostic groups were evaluated using estimated marginal means (emmeans) with a Tukey Honest Significant Difference (HSD) adjustment.

##### 2.4.1.3 Intra-individual analysis

We examined Spearman correlation between the Canonicity and Relatedness scores and also examined intra-individual patterns to determine if impairment in the Canonicity and Relatedness scores occurred simultaneously or independently. For each patient, Z-scores were computed based on the control group’s mean and standard deviation. A score was considered significantly impaired if the Z-score exceeded + 1.65 (one-tailed *p* < 0.05).

##### 2.4.1.4 Cognitive correlates

We used two exploratory methods to study the cognitive processes associated with the controlled-retrieval and semantic selections mechanisms of semantic control. First, we performed Spearman correlations between performance on the neuropsychological tests and the Canonicity (reflecting controlled-retrieval) and Relatedness (reflecting selection) scores within patients only (to avoid the confounding effects of frequent at ceiling scores in controls. The results were adjusted using FDR correction.

To better control for possible confounding effects of diagnosis, disease severity, and key demographic factors, we employed a second exploratory analysis which consisted in the construction of multiple regression models designed to directly test the hypotheses that controlled retrieval would be preferentially associated with tests of semantic knowledge and executive function, whereas semantic selection would correlate primarily with executive function. We fitted a separate robust linear regression model (MM-estimation) predicting either the Canonicity or the Relatedness score from each semantic (animal verbal fluency, synonym judgment, and Boston Naming Test) or executive (digit span backward, TMT-B, and phonemic fluency) neuropsychological test including diagnosis, age, sex, education years, and MMSE as covariates (total of 12 models). Given the exploratory nature of this analysis and the limited statistical power imposed by our sample size (*N* = 28 patients), results are reported without correction (Supplementary Table 2).

#### 2.4.2 Imaging correlates of performance on the semantic control task

*Whole brain analysis:* For each condition and score, we assessed the relationship with GM volume using voxel-wise multiple linear regression in SPM12 in patients. Analyses were performed in a mask that excluded non-GM voxels, and the statistical models were corrected for total intracranial volume (TIV). Statistical maps were thresholded at *p* < 0.005 (uncorrected) at the voxel level with a cluster extent threshold of > 100 mm^3^ (corresponding to 30 voxels at 1.5x1.5x1.5 mm resolution). Resulting T-maps were visualized using Surf Ice software for rendering, with color indicating T-statistics from the regression analysis.

**FIGURE 2:**
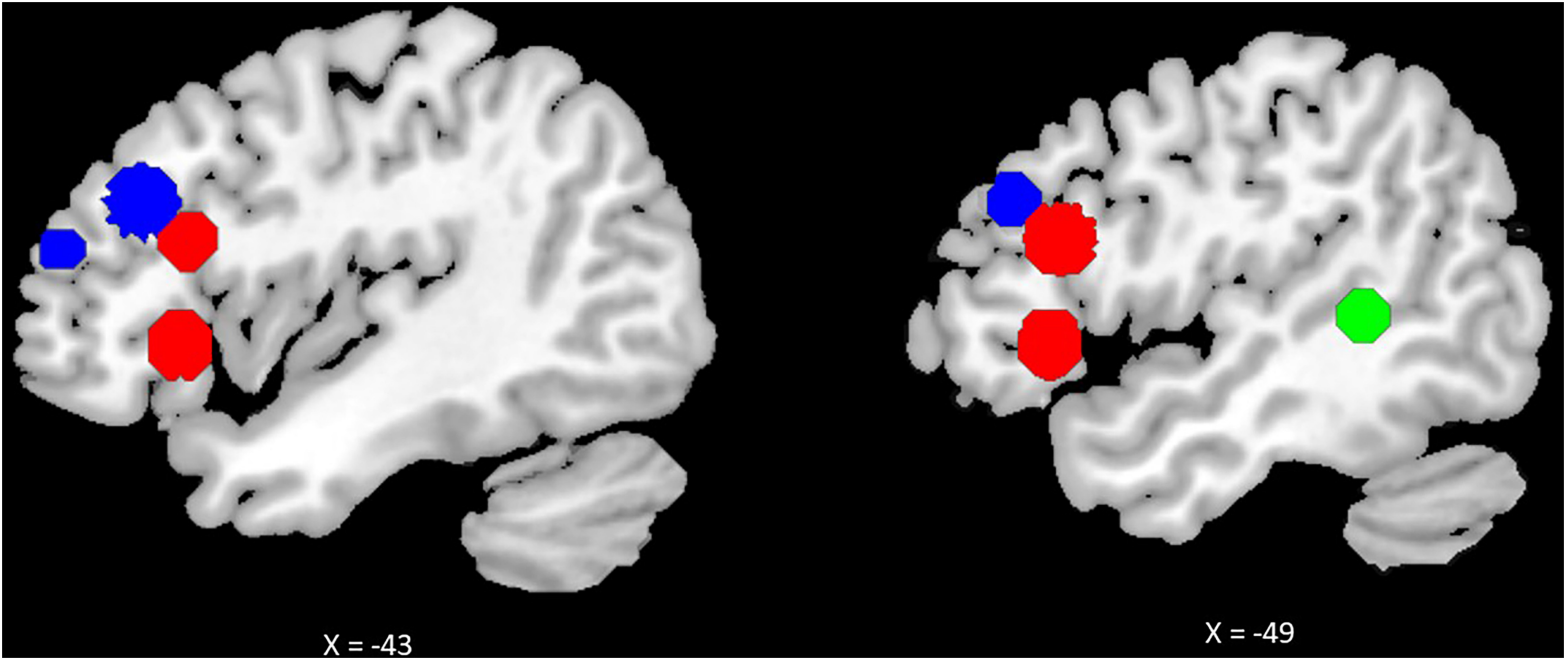
Anatomical localization of the a priori Regions of Interest (ROIs). The five spherical Hodgson ROIs used for the target analyses are overlaid on the standard ch2_better template. Because these target regions are distributed across different anatomical depths, they are presented across complementary slices (*X* = –43 and *X* = –49) to ensure full visual representation of all five volumes. MNI coordinates (X-axis) are provided for each slice, and distinct colors differentiate the specific regions evaluated: Red: left IFG (pars triangularis and pars orbitalis); Green: left pMTG; and Blue; left MFG anterior and mid).

*Region of interest (ROI) imaging analysis:* We conducted a targeted Region of Interest (ROI) analysis to test our hypothesis that controlled retrieval would be preferentially associated with atrophy in key areas of the semantic control network, such as the IFG and posterior middle temporal gyrus (pMTG), whereas semantic selection would correlate primarily with atrophy in frontal areas included in both the semantic and domain-general cognitive control systems, such as the IFG and middle frontal gyrus (MFG). Structural masks were derived from the established frameworks of Jackson (2021) for the semantic control network and Fedorenko et al. (2013) for the domain-general network. Analyses were conducted within the a priori ROI masks published by Hodgson et al. (2024), obtained directly from the study’s open-access GitHub repository. We selected only the left hemisphere ROIs to maintain anatomical consistency with the left-lateralized semantic control network and to evaluate how local, domain-general executive structures interact with the primary left-hemisphere semantic hubs. As documented by Hodgson et al. (2024), these open-source network templates show a partial overlap between the left MFG and IFG (pars triangularis) masks (Figure 2).

To evaluate brain-behavior associations, we constructed a series of linear regression models. First, we extracted the median GM volume from each ROI using the unsmoothed NIfTI images of the patient cohort. To determine whether structural associations varied by clinical phenotype, we built a model to predict the Canonicity and Relatedness scores by testing the interaction between regional GM volume and diagnostic group: Score ∼ GM_ROIxdiagnosis. Second, where interaction terms were non-significant, a simplified main-effects model was evaluated to examine the overall predictive value of regional atrophy across the patient cohort: score ∼ GM_ROI + diagnosis. In both models, age, gender, years of education, and total intracranial volume (TIV) were consistently incorporated as covariates. We used the False Discovery Rate (FDR) method to correct for multiple comparisons.

## 3 Results

### 3.1 Demographic and neuropsychological data

Demographic, neurological, and neuropsychological data for each diagnosis group are provided in Table 1. We found significant differences between the control group and both patient groups in age and education, with the control group being younger and more educated. As expected, significant differences were found for MMSE between both patient groups and the control group, but not between the patient groups. All neuropsychological measures showed significant differences between the control group and both patient groups, with the following exceptions: the sentence comprehension, digit span forward, sentence repetition, and Poppelreuter tests showed no significant differences between controls and ADtyp patients; the number location subtest from the VOSP Battery showed no significant differences between controls and lvPPA patients. The only significant differences observed between the ADtyp and lvPPA groups were in the Boston Naming Test (BNT), and short and long sentence repetition tests (with sentence comprehension, VOSP number location, and CERAD visual memory trending towards significance).

**TABLE 1:** Demographic data and raw neuropsychological scores per diagnosis group.

Measure	Control *N* = 16	ADtyp *N* = 11	lvPPA *N* = 17	*p* control vs. ADtyp	*p* control vs, lvPPA	*p* ADtyp vs. lvPPA
Demographics						
Age (years)	66.1 [60.5; 67.8]	70.9 [66.7; 76.2]	74.7 [68.3; 76.4]	**0.018**	**0.001**	0.438
Age onset (years)	-	66.2 [60.7; 70.9]	70.5 [64.4; 73.2]	-	-	0.246
Gender				1.000	0.858	0.638
Male	7 (43.8%)	4 (36.4%)	9 (52.9%)			
Female	9 (56.2%)	7 (63.6%)	8 (47.1%)			
Education (years)	17.5 [15.0; 20.0]	11.0 [9.5; 17.5]	12.0 [9.0; 16.0]	**0.026**	**0.002**	0.905
Global scale						
MMSE (max. score 30)	29.0 [28.8; 30.0]	24.0 [22.0; 25.0]	24.0 [19.0; 27.0]	**< 0.001**	**<0.001**	0.944
Language						
Boston naming test (max. score 60)	56.5 [53.8; 58.0]	45.0 [40.0; 48.0]	34.5 [20.2; 39.5]	**< 0.001**	**<0.001**	**0.009**
Synonyms (max. score 100)	95.0 [92.5; 97.5]	85.0 [75.0; 86.2]	85.0 [75.0; 87.5]	**< 0.001**	**<0.001**	0.887
Comprehension (max. score 15)	15.0 [15.0; 15.0]	15.0 [15.0; 15.0]	14.0 [12.2; 15.0]	0.644	**0.012**	0.051
Verbal fluency animals (# of animal names in 60 sec.)	35.0 [27.0; 42.0]	16.0 [13.5; 23.2]	14.0 [10.2; 18.0]	**0.002**	**< 0.001**	0.177
Sentence repetition short	175.0 [174.5; 175.5]	172.0 [159.5; 176.0]	101.0 [83.0; 144.0]	0.516	**0.007**	**0.001**
Sentence repetition long	314.0 [300.5; 327.0]	230.0 [135.0; 297.0]	94.0 [60.0; 135.0]	0.052	**0.007**	**0.002**
Executive function						
Verbal fluency “p” words (# of words in 60 sec.)	26.0 [22.8; 29.8]	16.5 [10.2; 23.8]	14.5 [7.5; 16.0]	**0.008**	**< 0.001**	0.394
TMT_A (time to complete in sec.)	41.0 [32.5; 46.8]	62.5 [46.0; 116.0]	59.0 [45.0; 127.5]	**0.012**	**0.002**	0.860
TMT_B (time to complete in sec.)	82.0 [64.5; 105.5]	650.0 [489.5; 700.0]	205.5 [159.8; 700.0]	**< 0.001**	**<0.001**	0.470
Digit span forwards	6.0 [5.0; 7.0]	5.0 [4.2; 5.8]	4.0 [3.0; 5.0]	0.191	**0.010**	0.133
Digit span backwards	5.0 [4.0; 5.0]	3.0 [3.0; 4.0]	3.0 [3.0; 3.0]	**0.001**	**< 0.001**	0.349
Visuo-spatial/ visuopercetpive/ visuoconstructional function						
Number location VOSP (max. score 10)	9.0 [7.0; 10.0]	5.5 [2.8; 7.8]	8.0 [5.2; 9.0]	**0.004**	0.137	0.066
Clock drawing test (max. score 10)	9.8 [7.9; 10.0]	6.0 [4.2; 7.2]	5.5 [4.0; 7.0]	**0.005**	**0.002**	0.803
CERAD figures copy (max. score 11)	11.0 [11.0; 11.0]	9.0 [7.5; 10.0]	10.5 [9.0; 11.0]	**< 0.001**	**0.017**	0.152
Poppelreuter (max. score 5)	10.0 [10.0; 10.0]	10.0 [9.2; 10.0]	10.0 [9.0; 10.0]	0.101	**0.040**	0.649
Visual memory						
CERAD figures recall (max. score 11)	10.0 [8.0; 11.0]	0.0 [0.0; 0.8]	3.5 [0.0; 8.0]	**< 0.001**	**0.001**	0.075
Verbal memory						
FCSRT immediate free recall (max. score 48)	29.5 [26.8; 32.2]	4.0 [2.2; 12.0]	9.0 [4.0; 13.0]	**< 0.001**	**<0.001**	0.383
FCSRT immediate cued recall (max. score 48)	46.5 [46.0; 47.2]	16.5 [13.2; 22.8]	23.0 [10.0; 29.0]	**< 0.001**	**<0.001**	0.385
FCSRT delayed free recall (max. score 16)	12.5 [11.0; 14.0]	0.0 [0.0; 2.2]	2.0 [0.0; 6.0]	**< 0.001**	**<0.001**	0.141
FCSRT delayed cued recall (max. score 16)	16.0 [16.0; 16.0]	5.5 [2.8; 7.8]	10.0 [3.0; 11.0]	**< 0.001**	**<0.001**	0.224
n(%); median [25th; 75th]						

Continuous variables were compared across the three groups using the Kruskal-Wallis test for overall comparisons followed by Wilcoxon rank-sum test for *post-hoc* pairwise between-group comparisons. Categorical distributions (gender) were evaluated using a Chi-squared test for the overall group comparison, followed by pairwise Fisher’s exact tests for *post-hoc* between-group comparisons. *P*-values < 0.05 (in bold) were considered statistically significant. MSE, Mini-Mental State Examination (Blesa et al., 2001); BNT, Boston Naming Test (Spanish-language version (García-Albea et al.,, 1996); Synonyms (Noonan et al., 2010); Comprehension: order comprehension from CERAD battery (Morris et al., 1988); Verbal fluency animals; Sentence repetition tests (Goodglass et al., 1986); Verbal fluency “p” words (fluency with p cue) (Peña-Casanova et al., 2009b); TMT, Trail-Making Test (Peña-Casanova et al., 2009b); Digit span forwards, Digit span backwards (Peña-Casanova et al., 2009b); Clock Drawing task (Cacho et al., 1996); VOSP, Visual Object and Space Perception Battery (Peña-Casanova et al., 2009c); Figures CERAD (Morris et al., 1988); FCSRT, Free and Cued Selective Reminding Test (Peña-Casanova et al., 2009a); Poppelreuter Overlapping Figures Test (Poppelreuter, 1917).

### 3.2 Between-group comparison of task accuracy

Pairwise comparisons of global accuracy revealed an overall reduction in performance in the lvPPA relative to the control group (OR = 2.24, 95% CI [1.07, 4.69], Tukey-adjusted *p* = 0.029), whereas the ADtyp group did not differ significantly from controls (OR = 1.69, 95% CI [0.78, 3.77], Tukey-adjusted *p* = 0.2254) or lvPPA groups (OR = 1.33, 95% CI [0.69, 2.55], Tukey-adjusted *p* = 0.571) (Table 2).

**TABLE 2:** Pairwise diagnostic group contrasts from the interaction model.

Contrast	Odds ratio	Lower 95% CI	Upper 95% CI	*p*-value
Control/ADtyp	1.69	0.78	3.67	0.254
Control/lvPPA	2.24	1.07	4.69	**0.029**
ADtyp/lvPPA	1.33	0.69	2.55	0.571

Data are presented as odds ratios (effect sizes) with corresponding 95% confidence intervals (CI). *p*-values and confidence limits are adjusted for multiple comparisons using the Tukey method. Bold values indicate statistically significant differences (*p* < 0.05).

Pairwise contrasts of accuracy in each experimental condition across diagnostic groups revealed no significant differences between any of the groups during the low-demand condition (C-U, all *p* ≥ 0.251) or the canonical related condition (C-R, all *p* ≥ 0.278). For the noncanonical unrelated condition (N-U), healthy controls significantly outperformed the lvPPA cohort (Tukey-adjusted *p* = 0.007), whereas the ADtyp cohort did not significantly differ from controls (Tukey-adjusted *p* = 0.186) or the lvPPA group (Tukey-adjusted *p* = 0.392). For the most demanding condition, noncanonical related (N-R), healthy controls significantly outperformed both the ADtyp cohort (Tukey-adjusted *p* = 0.021) and the lvPPA cohort (Tukey-adjusted *p* = 0.008). Direct pairwise comparisons between the two patient groups were not significant under any of the conditions (Table 3).

**TABLE 3:** Logistic generalized linear mixed model predicting accuracy at the trial level.

	Correctness		
Predictors	Odd ratios	Confidence interval (CI)	*p*
Relatedness [related]	0.23	0.14–0.37	<0.001
Canonicity [noncanonical]	0.18	0.11–0.30	<0.001
Diagnosis [ADtyp]	0.89	0.38–2.05	0.779
Diagnosis [lvPPA]	0.54	0.25–1.16	0.113
Age	1.08	0.84-1.39	0.56
Sex[female]	0.97	0.63–1.49	0.895
Education years	1.52	1.20–1.95	**0.001**
Relatedness [related]: Canonicity [noncanonical]	1.61	1.04–2.49	**0.032**
Canonicity[noncanonical]:DX[ADtyp]	0.59	0.34–1.00	0.052
Canonicity[noncanonical]:DX[vlPPA]	0.54	0.25–1.16	0.069
Relatedness [related]: DX [ADtyp]	0.76	0.45–1.29	0.307
Relatedness [related]: DX [lvPPA]	1.08	0.1.72	0.741

Relatedness, canonicity, diagnosis group and education years act as predictors, and participant, action and target screen position as random intercepts: correct response ∼ Relatedness × Diagnosis group + Canonicity × Diagnosis group + Relatedness × Canonicity + Diagnosis group + Age + Sex + Education years + (1| participant) + (1| action) + (1| correct position). The reference levels for the categorical predictors were: unrelated (for relatedness), canonical (for canonicity), and control (for diagnostic group). Bold values indicate statistically significant differences (*p* < 0.05).

We evaluated the experimental task data quality and found eight trials in total that fulfilled one or both of the criteria indicated in the methods to be considered as problematic [8 for (i) and 2 for (ii), see Supplementary material section 1]. Their exclusion did not affect the overall results, therefore we report the results that take into account all trials.

### 3.3 Evaluation of the effects of the Canonicity and Relatedness experimental manipulations

To understand how varying levels of canonicity and relatedness impact each diagnosis group, four different analyses were conducted.

**FIGURE 3:**
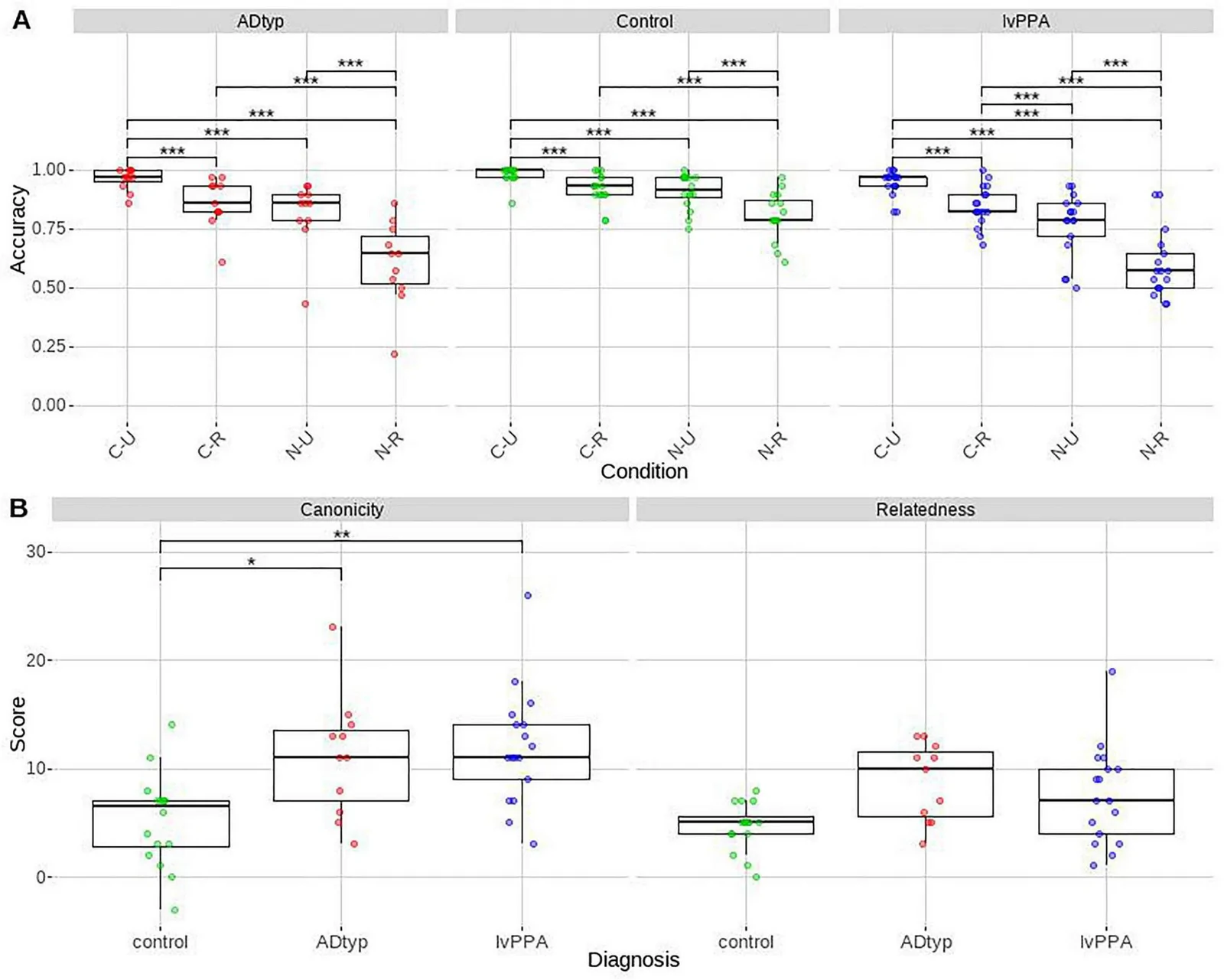
Accuracy for each condition and experimental score across diagnostic groups. Boxplots show group distributions; colored points represent individual accuracy and scores by diagnostic group. All pairwise statistical significances are reported using Tukey-adjusted *p*-values ns: *p* > 0.05; *p ≤ 0.05; **p ≤ 0.01; ***p ≤ 0.001. **(A)** Accuracy per condition [canonical unrelated (C-U); canonical related (C-R); noncanonical unrelated (N-U); and noncanonical related (N-R)] and diagnosis group (control, ADtyp, lvPPA). Overlaid significance brackets correspond to demographic-adjusted, *post-hoc* within-group pairwise comparisons derived from the generalized logistic mixed-effects trial-level model of task accuracy. **(B)** Canonicity and Relatedness scores per diagnostic group. Overlaid significance brackets correspond to *post-hoc* between-group pairwise comparisons derived from two separate multiple linear regression models, one for each score, controlling for age, sex, and years of education.

First, *post-hoc* within-group pairwise comparisons were derived from the optimal trial-level accuracy model, fully adjusted for age, sex, and education. Global accuracy reached ceiling levels ( ≥ 90%) only for the control group. Within each diagnostic group, accuracy decreased as the cognitive control demands of the task condition increased (Figure 3A). Significant differences (Tukey-adjusted *p* ≤ 0.0001) were observed between all pairs of conditions for the three groups, except between the canonical related and noncanonical unrelated conditions for the control and ADtyp groups (Tukey-adjusted *p* = 0.836 for controls; Tukey-adjusted *p* = 0.228 for ADtyp) (Figure 3A).

Second, interpretation of the optimal trial-level accuracy model (Table 4) indicated both experimental manipulations work as expected, noncanonical targets (OR = 0.18, *p* < 0.001) and related distractors (OR = 0.23, *p* < 0.001) both strongly reduce accuracy. However the significant interaction term Relatedness × Canonicity (OR = 1.61, *p* = 0.032) means the combined difficulty of the N-R condition (noncanonical target + related distractors) is less severe than expected from adding each factor’s main effects, suggesting there is a limit to how much harder the task can get. The Odds ratios of both the Canonicity × Diagnosis[ADtyp] (OR = 0.59, *p* = 0.052) and Canonicity × Diagnosis [lvPPA] (OR = 0.54, *p* = 0.069) interaction terms indicate both patient groups show decreased probability of a correct answer for noncanonical targets compared to controls, though both were only marginally significant possibly due to a power issue given the small sample size. In contrast, the Relatedness x Diagnosis interaction for both patient groups ([ADtyp]: OR = 0.76, *p* = 0.307; [lvPPA]: OR = 1.08, *p* = 0.741) was clearly non-significant suggesting neither patient group showed a significantly larger relatedness effect than controls at the trial level.

**TABLE 4:** Global accuracy, accuracy per condition, and scores per diagnosis group.

Measure	control *N* = 16	ADtyp *N* = 11	lvPPA *N* = 17	p.control vs. ADtyp	p.control vs. lvPPA	p.ADtyp vs. lvPPA
Accuracy	0.91 [0.90; 0.94]	0.84 [0.77; 0.86]	0.79 [0.72; 0.83]	0.254	**0.029**	0.571
Accuracy C-U	1.00 [0.96; 1.00]	0.96 [0.95; 1.00]	0.96 [0.93;0 .96]	0.957	0.251	0.347
Accuracy C-R	0.93 [0.89; 0.96]	0.86 [0.82; 0.93]	0.82 [0.82; 0.89]	0.555	0.278	0.886
Accuracy N-U	0.91 [0.88; 0.96]	0.86 [0.79; 0.89]	0.79 [0.71; 0.86]	0.186	**0.007**	0.392
Accuracy N-R	0.79 [0.79; 0.87]	0.64 [0.52; 0.71]	0.57 [0.50; 0.64]	**0.021**	**0.008**	0.978
Canonicity score	6.50 [2.75; 7.00]	11.00 [7.00; 13.50]	11.00 [9.00; 14.00]	**0.023**	**0.004**	0.814
Relatedness score	5.00 [4.00; 5.50]	10.00 [5.50; 11.50]	7.00 [4.00; 10.00]	0.227	0.577	0.691
Median [25th; 75th]						

Conditions: canonical unrelated (C-U); canonical related (C-R); noncanonical unrelated (N-U); and noncanonical related (N-R). The median and interquartile range (25th and 75th percentiles) are displayed. *Post-hoc* pairwise comparisons for accuracy metrics across diagnostic groups derived from the optimal generalized logistic mixed model of trial-level task accuracy, whereas comparisons for Canonicity and Relatedness scores are derived from independent multiple linear regressions. *P*-values are corrected for multiple comparisons using Tukey’s method. Bold values indicate statistically significant differences (*p* < 0.05).

Third, to further assess the effect of the Canonicity and Relatedness manipulations on accuracy, and if this effect differed according to diagnosis, we also conducted condition-specific pairwise group comparisons decomposing the diagnosis × canonicity and diagnosis x relatedness interactions. For the canonical condition, no significant group differences were observed (all *p*_values > 0.23). In contrast, for the noncanonical condition, controls showed higher accuracy than both the ADtyp (OR = 2.20, 95% CI [1.01, 4.82], *p* = 0.047) and lvPPA (OR = 2.79, 95% CI [1.32, 5.91], *p* = 0.004) groups, whereas the ADtyp and lvPPA groups did not differ (OR = 1.27, 95% CI [0.65, 2.46], *p* = 0.683). Thus, both patient groups showed worse performance for noncanonical items (reflecting impairment in controlled-retrieval), whereas performance was comparable across groups for canonical items (Table 5). For unrelated items, the control group showed higher accuracy than the lvPPA group (OR = 2.33, 95% CI [1.02, 5.31], *p* = 0.043), whereas differences between controls and the ADtyp group (OR = 1.47, 95% CI [0.61, 3.55], *p* = 0.559) and between the ADtyp and lvPPA groups (OR = 1.58, 95% CI [0.75, 3.33], *p* = 0.320) were not significant. A similar pattern was observed for related items, where the control group again outperformed the lvPPA group (OR = 2.15, 95% CI [1.01, 4.58], *p* = 0.046), while the remaining comparisons were not significant. Thus, the lvPPA group showed impairment in both unrelated (low semantic selection demand) and related (high selection demand) conditions, whereas ADtyp did not show significant impairment in either (Table 5).

**TABLE 5:** Condition-specific *post-hoc* pairwise comparisons of performance accuracy by diagnostic group.

Condition	Contrast	Odds ratio	Lower 95% CI	Upper 95% CI	*p*.value
Canonicity					
Canonical	Control/ADtyp	1.29	0.53	3.16	0.779
	Control/lvPPA	1.79	0.78	4.14	0.229
	ADtyp/lvPPA	1.39	0.65	2.97	0.569
Noncanonical	Control/ADtyp	2.20	1.01	4.82	**0.047**
	Control/lvPPA	2.79	1.32	5.91	**0.004**
	ADtyp/lvPPA	1.27	0.65	2.46	0.683
Relatedness					
Unrelated	Control/ADtyp	1.47	0.61	3.55	0.559
	Control/lvPPA	2.33	1.02	5.31	**0.043**
	ADtyp/lvPPA	1.58	0.75	3.33	0.320
Related	Control/ADtyp	1.93	0.88	4.26	0.122
	Control/lvPPA	2.15	1.01	4.58	**0.046**
	ADtyp/lvPPA	1.11	0.57	2.18	0.927

Results display the decomposition of the Diagnosis × Canonicity and Diagnosis × Relatedness interaction terms from the final trial-level generalized linear mixed model. Pairwise group contrasts are presented across stimulus conditions, adjusted for demographic covariates (age, sex, education) using Tukey-adjusted *p*-values. Bold values indicate statistically significant differences (p<.05).

Fourth, the Canonicity and Relatedness scores were compared across diagnostic groups via multiple linear regression adjusting for demographic covariates. *Post-hoc* Tukey-adjusted pairwise contrasts demonstrated that healthy controls exhibited significantly lower impairment scores compared to both ADtyp (β = –5.48, SE = 1.98, *p* = 0.023) and lvPPA (β = –6.53, SE = 1.89, *p* = 0.004). Direct comparisons between the two patient groups revealed no statistically significant difference (β = –1.05, SE = 1.71, *p* = 0.814). For the Relatedness score, controls did not differ significantly from either ADtyp (β = –2.72, SE = 1.62, *p* = 0.227) or lvPPA (β = –1.56, SE = 1.55, *p* = 0.577). Direct contrasts between the two patient groups showed no significant differences (β = 1.16, SE = 1.41, *p* = 0.691) (Figure 3B).

### 3.4 Intra-individual pattern of impairment in canonicity and relatedness scores

**FIGURE 4:**
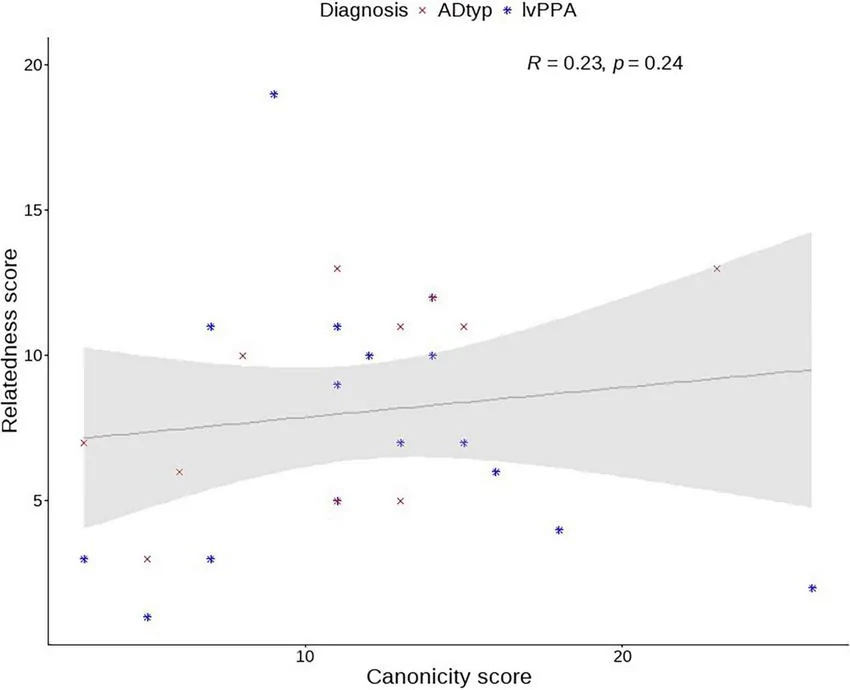
Spearman correlation between Canonicity and Relatedness scores (r_s_, *p*-values). Individual scores of ADtyp patients are shown as red crosses and lvPPA patients as blue stars.

**FIGURE 5:**
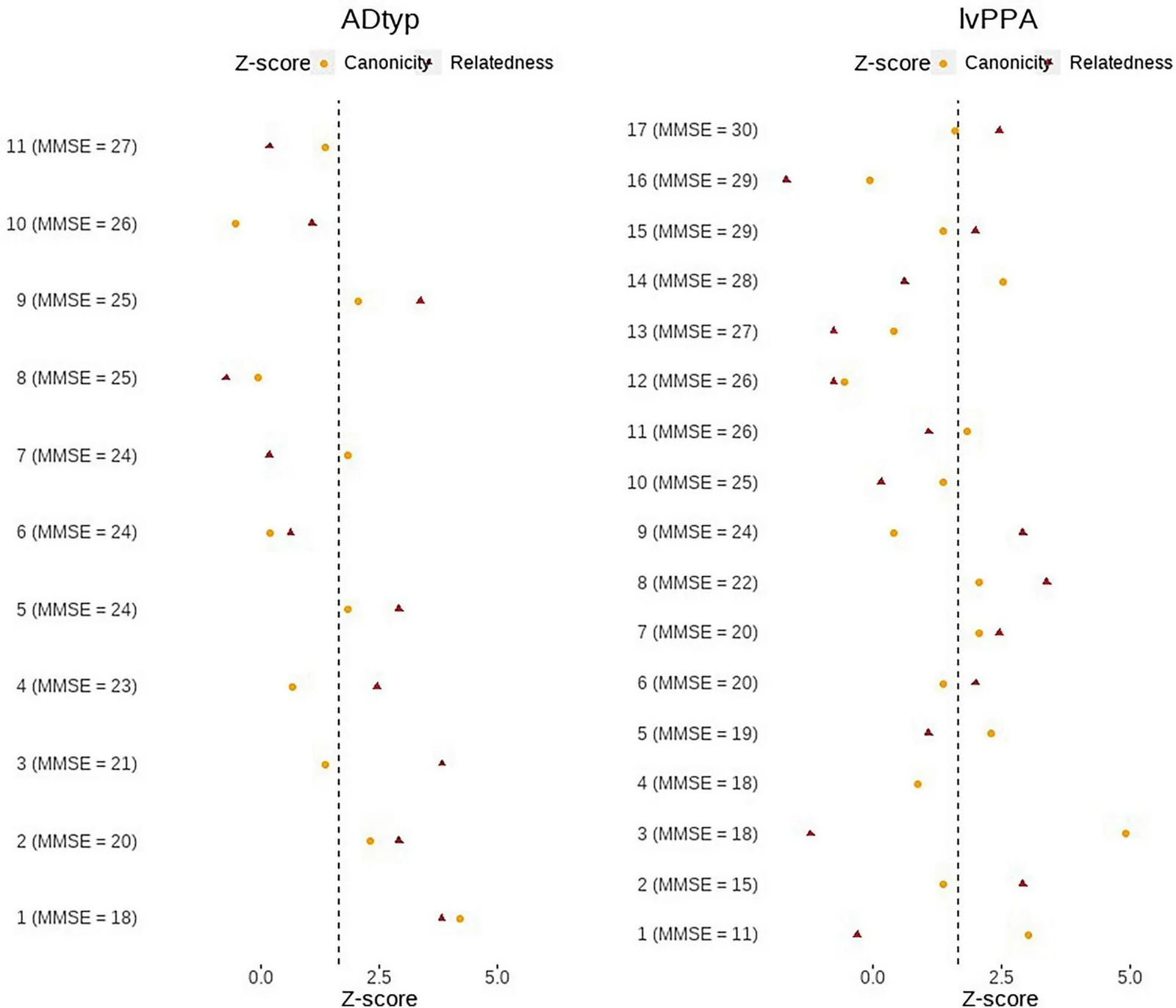
Individual experimental Z-scores and correlations between experimental scores. Canonicity and relatedness, Z-scores per patient. Canonicity score is shown as orange points, Relatedness score as brown triangles. Dashed line at 1.65 standard deviations from the control mean. Within each patient group, patients are sorted according to their MMSE score.

Canonicity and Relatedness scores did not correlate (*p* = 0.24) in patients (Figure 4). We examined whether impairment in the Canonicity and Relatedness scores tended to occur in a concomitant or dissociated manner. As displayed on Figure 5, among the subset of 20 patients presenting significant impairment (Z-score beyond 1.65) in either Canonicity or Relatedness scores, only six presented concurrent impairment in both.

### 3.5 Cognitive correlates of the canonicity and relatedness scores

We performed a correlation analysis between the experimental task and neuropsychological scores, taking into account patients only to avoid spurious associations related to the ceiling effects present in the control group (Table 6). No correlations survived FDR correction. At an uncorrected threshold, the Canonicity score correlated significantly with the MMSE and long sentence repetition scores, and showed a marginal correlation with synonyms and animal verbal fluency scores. The Relatedness score correlated with the MMSE, digit span forward and backward, and TMT B scores. We also fitted separate robust linear regression models predicting either the Canonicity or the Relatedness score from each semantic (animal verbal fluency, synonym judgment, and Boston Naming Test) or executive (digit span backward, TMT-B, and phonemic fluency) neuropsychological test including diagnosis, age, sex, education years, and MMSE as covariates (total of 12 models). The results revealed that, after controlling for age, education, sex, diagnosis, and MMSE, animal verbal fluency was the only semantic measure to significantly predict the Canonicity score (β = –2.21, 95% CI [–3.42, –0.99], *p* = 0.001), with lower fluency scores associated with higher (more impaired) Canonicity scores. Neither synonym judgment nor BNT score predicted canonicity (all *p* > 0.81). For the Relatedness score, three measures reached significance after adjustment for the same covariates: animal verbal fluency (β = –1.52, 95% CI [–2.40, –0.63], *p* = 0.002), digit span backward (β = –1.70, 95% CI [–2.93, –0.46], *p* = 0.010), and phonemic fluency (β = –1.24, 95% CI [–2.47, –0.02], *p* = 0.047). TMT-B and synonym judgment did not significantly predict the Relatedness score (all *p* > 0.22) (Supplementary Table 2).

**TABLE 6:** Spearman correlation between scores and neuropsychological tests, patients only.

	Canonicity score				Relatedness score			
Test	*N*	*r*	*p* (uncor.)	*p* (FDR)	*N*	*r*	*p* (uncor.)	*p* (FDR)
Global scale								
MMSE	28	–0.44	**0.020**	0.277	28	–0.37	**0.050**	0.285
Language								
Boston naming test	24	–0.09	0.675	0.787	24	–0.16	0.463	0.627
Synonyms	28	–0.37	0.054	0.285	28	–0.26	0.185	0.432
Comprehension	24	–0.36	0.089	0.310	24	–0.33	0.117	0.342
Verbal fluency animals	24	–0.40	0.052	0.285	24	–0.36	0.086	0.310
Sentence repetition short	28	–0.35	0.065	0.304	28	–0.16	0.405	0.594
Sentence repetition long	28	–0.46	**0.014**	0.277	28	–0.24	0.228	0.504
Executive function								
Verbal fluency “p” words	24	–0.16	0.449	0.627	24	–0.34	0.105	0.339
TMT A	24	0.18	0.388	0.594	24	0.23	0.283	0.567
TMT B	24	0.12	0.565	0.698	24	0.41	**0.046**	0.285
Digit span forward	24	–0.19	0.378	0.594	24	–0.53	**0.008**	0.277
Digit span backward	24	–0.18	0.392	0.594	24	–0.44	**0.032**	0.285
Visuospatial/visuopercetpive/visuoconstructional function								
Number location VOSP	24	–0.08	0.722	0.798	24	–0.03	0.885	0.885
Clock drawing test	23	–0.19	0.392	0.594	23	–0.33	0.122	0.342
CERAD figures copy	20	0.09	0.693	0.787	20	0.06	0.787	0.806
Poppelreuter	24	–0.29	0.173	0.432	24	–0.36	0.086	0.310
Visual memory								
CERAD figures recall	20	0.07	0.761	0.806	20	–0.11	0.635	0.762
Verbal memory								
FCSRT immediate free recall	24	0.15	0.481	0.627	24	–0.24	0.256	0.538
FCSRT immediate cued recall	24	0.18	0.403	0.594	24	–0.19	0.385	0.594
FCSRT delayed free recall	24	0.28	0.182	0.432	24	0.06	0.779	0.806
FCSRT delayed cued recall	24	0.18	0.410	0.594	24	–0.15	0.493	0.627

*P*-values unadjusted and FDR corrected (< 0.05 in bold, < 0.06 underlined).

### 3.6 Gray matter volume comparison across diagnostic groups

**FIGURE 6:**
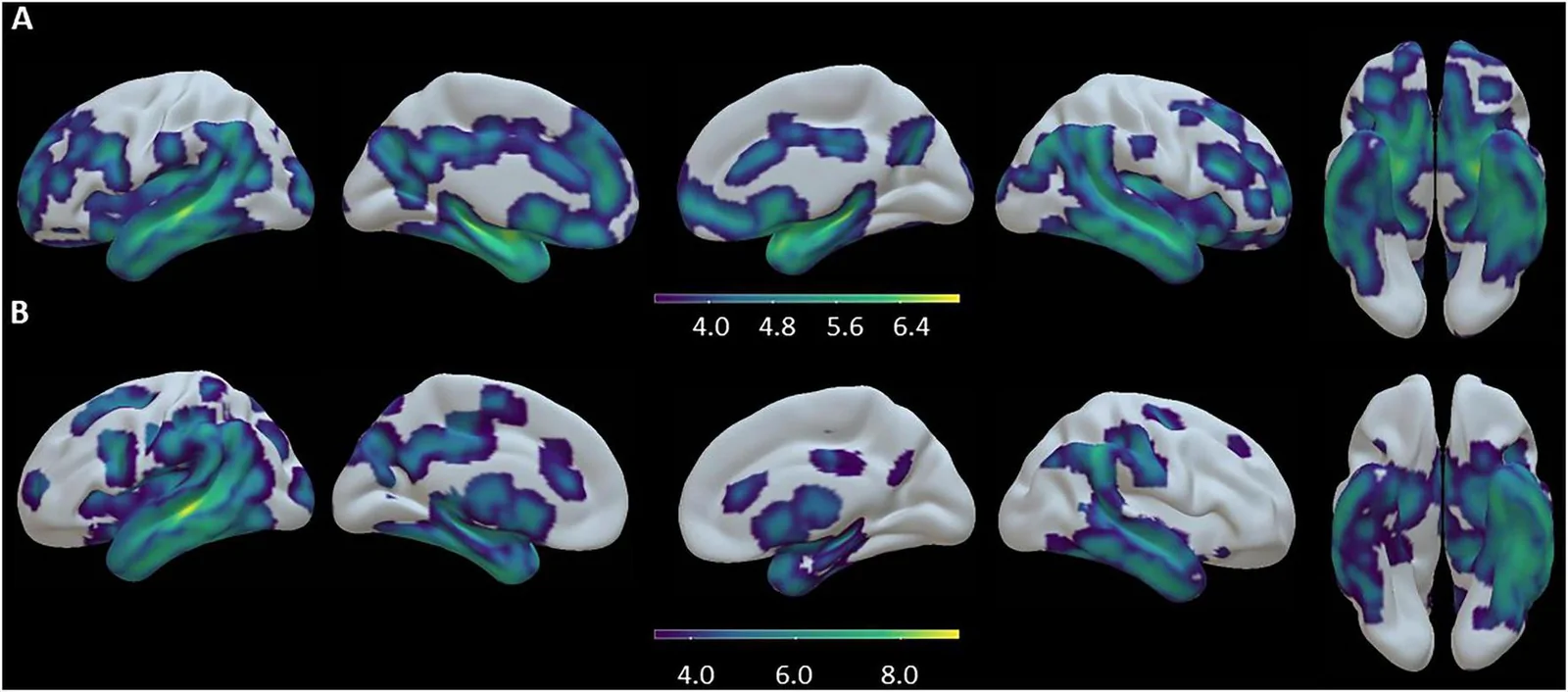
Decreased gray matter volume in ADtyp **(A)** and lvPPA **(B)** compared to the control group. Total intracranial volume was included as a covariate and maps are thresholded at *p* < 0.001 uncorrected with a cluster extent threshold of ∼ 100 mm^3^. Colors indicate *T*-values, with warmer colors reflecting stronger group differences. Surface projections were generated using Surf Ice software.

Between-group comparisons revealed significant differences in GM volume between the control group and both patient groups (Figure 6). Specifically, the volume loss in ADtyp predominated in the medial and lateral temporal lobe and further encompassed parietal structures, the cingulate and dorsolateral frontal regions. In lvPPA, the volume loss involved similar brain regions but predominated in the left mid-posterior temporal lobe and showed a clear asymmetry. No significant results were found when comparing GM volume in lvPPA vs. ADtyp.

### 3.7 Gray matter correlates of performance on the experimental task

**FIGURE 7:**
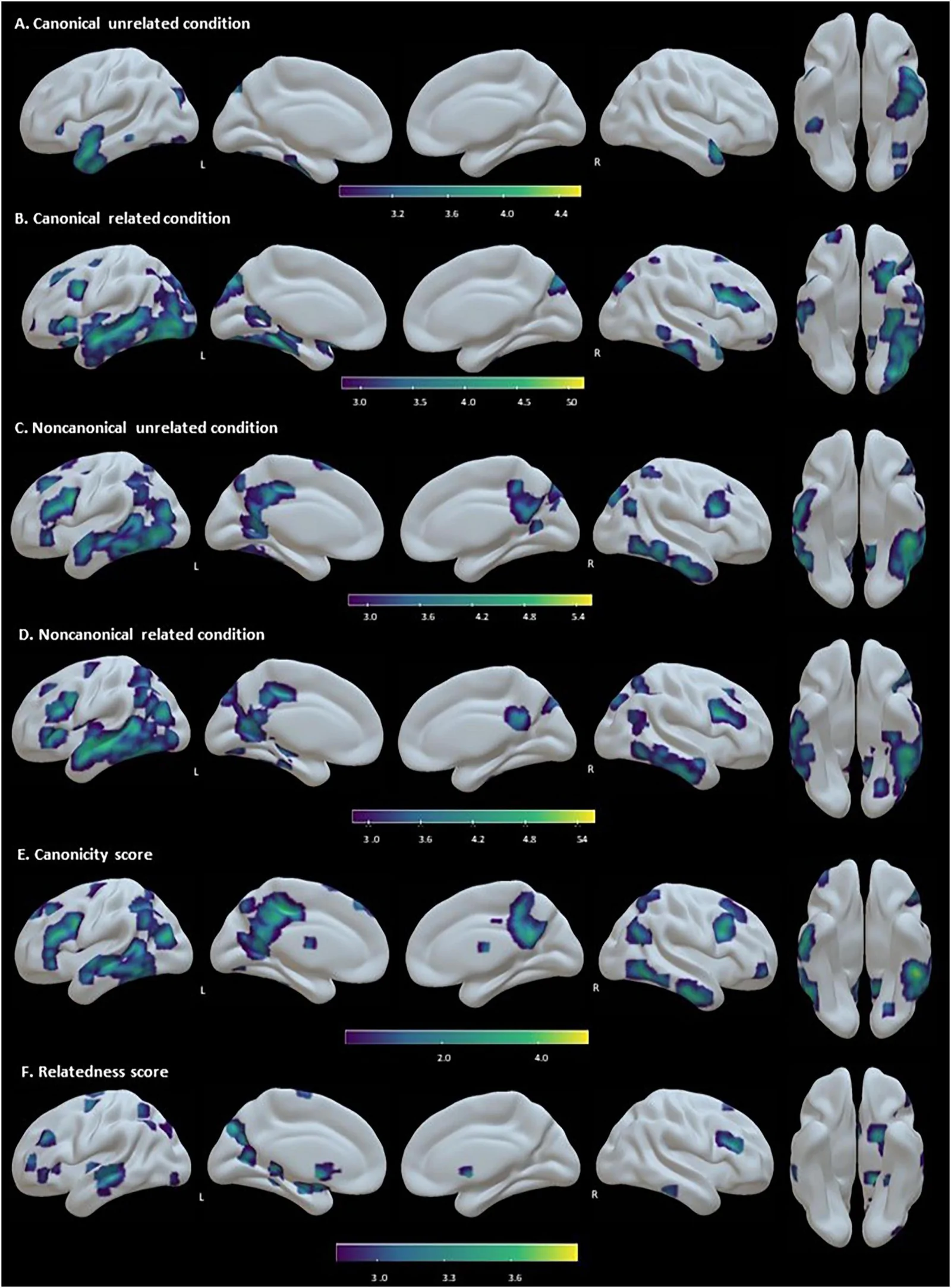
GM volume and accuracy. Left column **(A–G)** Correlation of GM volume with accuracy under each of the 4 conditions for patients. Total intracranial volume was included as a covariate and maps were thresholded at *p* < 0.005 uncorrected at the voxel level with a cluster extent threshold of ∼ 100 mm^3^. The color bar reflects *t*-values from the voxel-wise statistical analysis: **(A)** canonical unrelated condition; **(B)** canonical related condition; **(C)** noncanonical unrelated condition; and **(D)** noncanonical related condition; and with the 2 scores: **(E)** Canonicity score; and **(F)** Relatedness score.

We performed correlation of patients’ GM volume with accuracy under each of the 4 conditions and with the Canonicity and Relatedness scores (Figure 7) (*p* < 0.005, *k* > 30. GM correlation with total accuracy is shown in Supplementary Figure 1. Due to the sample size of our patient cohort (*N* = 25), the exploratory voxel-wise neuroimaging analysis was adjusted only for total intracranial volume (TIV). Consequently, the imaging results should be treated as descriptive. While several key clusters survived strict family-wise error (FWE) corrections, the remaining non-significant contrasts effects must be interpreted with caution. Given our modest sample size, the sensitivity to smaller, sub-threshold structural effects is reduced.

Accuracy in the canonical unrelated condition correlated with GM in the bilateral anterior temporal lobe (ATL). The other conditions produced correlates in frontal and posterior areas of the temporal and parietal lobes. Specifically, performance under the canonical related condition showed significant correlation with GM volume in the left fusiform gyrus (FG), posterior inferior temporal gyrus, pMTG, and left inferior and middle occipital gyri. Further correlations were also found in the left angular gyrus, orbitofrontal cortex, inferior frontal gyrus (IFG), insula, inferior parietal lobe (IPL), right inferior temporal gyrus (ITG), as well as bilaterally in the MFG. The non-canonical unrelated condition showed correlations in all of the areas mentioned above for the canonical related condition, plus bilaterally in the precuneus and posterior and middle cingulate cortex, and left supplementary motor area. The non-canonical related condition showed correlations overlapping with the canonical related condition, and further encompassing the IFG, angular gyrus, precuneus, posterior and middle cingulate cortex, and precentral gyrus.

The Canonicity and Relatedness scores shared some common neural correlates, but also involved distinct brain regions. The Canonicity score showed significant correlation with GM in the left IFG, FG, pMTG, and lateral occipital cortex and in the bilateral ATL, MFG, superior frontal gyrus, ITG, angular gyrus, precuneus and posterior and middle cingulate cortex. The Relatedness score showed less extensive correlations predominantly in the left IFG, pMTG and precuneus, and bilateral MFG.

### 3.8 ROI analysis

We constructed separate linear regression models evaluating the interaction between regional GM volume and diagnostic group (GM_ROIxdiagnosis) for each ROI (left IFG, MFG, and pMTG, Figure 3) exclusively within the patient cohort (*N* = 25). We found no statistically significant interaction effects across any of the tested regions of interest indicating the relationship between structural cortical volume and task performance did not differ significantly between the ADtyp and lvPPA phenotypes.

Consequently, we evaluated the main-effects models (GM_ROI + diagnosis) with age, gender, education years, and TIV as covariates. For Canonicity, GM atrophy in all three targeted ROIs remained a robust predictor of impairment scores (Table 7). A significant negative effect was observed for the left IFG (β = –29.27, 95% CI = [–43.53, –15.01], FDR-adjusted *p* = 0.0013), the left pMTG (β = –37.86, 95% CI = [–70.13, –5.60], FDR-adjusted *p* = 0.024), and the left MFG (β = –34.80, 95% CI = [–53.85, –15.74], FDR-adjusted *p* = 0.0018). Parallel multiple linear regression analyses evaluating the Relatedness score revealed a significant negative association only within the left pMTG (β = –28.12, 95% CI = [–49.65, –6.60], FDR-adjusted *p* = 0.040). In contrast, associations within the left IFG or left MFG failed to reach statistical significance (Table 7).

**TABLE 7:** Main effects of ROIs GM volumes on canonicity and relatedness scores.

ROI	Estimate	Lower 95% CI	Upper 95% CI	t_value	*p* (uncor.)	*p* (FDR)
Canonicity						
Left IFG	–29.27	–43.53	–15.01	–4.31	0.000	0.001
Left pMTG	–37.86	–70.13	–5.60	–2.47	0.024	0.024
Left MFG	–34.80	–53.85	–15.74	–3.84	0.001	0.002
Relatedness						
Left IFG	–10.07	–23.12	2.98	–1.62	0.122	0.122
Left pMTG	–28.12	–49.65	–6.60	–2.74	0.013	0.040
Left MFG	–14.21	–30.39	1.97	–1.84	0.082	0.122

CI, 95% confidence interval; FDR, False Discovery Rate correction for multiple comparisons.

## 4 Discussion

Previous research in healthy aging cohorts has identified two dissociable forms of semantic control, controlled retrieval of knowledge and selection among competing representations, yet their status and the neural correlates in AD remain poorly characterized. To address this gap, we recruited individuals with ADtyp, lvPPA, and healthy controls, and administered an adapted version of the canonicity and distraction in object selection task (Corbett et al., 2011), manipulating controlled retrieval and semantic selection demands. We found that increased controlled-retrieval (reflected by the Canonicity manipulation) and semantic selection (reflected by the Relatedness manipulation) demands worsened performance in all groups, including controls. Various analyses converged suggesting that both patient groups displayed greater sensitivity to the canonicity manipulation (indicating worse controlled-retrieval) compared to controls, while neither showed a statistically significant additional relatedness effect. Furthermore, lvPPA scored consistently lower than the other groups in all conditions of the experimental task, even the condition with the lowest semantic control demands, suggesting greater presence of other types of semantic impairment such as control impairment uncoupled from the controlled-retrieval and selection mechanism demands of the task or impairment of semantic representations. Consistent with prior work, controlled-retrieval (canonicity) and selection (relatedness) scores showed partially differentiable cognitive associations, though these exploratory relationships must be interpreted with caution and require replication in larger cohorts as most associations did not survive multiple-comparison correction. Specifically, the Canonicity score showed stronger associations to semantic-knowledge tests, whereas the Relatedness score showed more associations with tests of executive function. In line with recent descriptions of the Controlled Semantic Cognition framework (Ralph et al., 2017; Jackson, 2021), the exploratory whole-brain neuroimaging results revealed a differential shift: at an uncorrected threshold, trials with low control demands mainly rely on anterior temporal regions, while trials with higher-control demands relied on broader frontal, posterior-lateral temporal, and parietal cortices. The Canonicity and Relatedness scores showed overlapping and distinct neuroanatomical correlates, potentially reflecting differing reliance on semantic versus domain-general control networks, though these unadjusted spatial patterns require cautious replication. Taken together, these results advance our understanding of how AD disrupts semantic cognition and refine its clinical characterization potentially informing improvements in diagnosis and symptom management strategies.

### 4.1 Controlled retrieval demands affected both patient groups more than controls

Semantic control demands significantly affected performance in all diagnostic groups, including healthy controls. Within-group comparisons revealed decreasing accuracy with increasing control demands across all four conditions in all diagnostic groups and the trial-level accuracy model indicated both the Canonicity and Relatedness manipulations significantly worsened performance whereas age had no effect. This finding aligns with previous studies showing the effects of semantic control demands in healthy aging cohorts (Hoffman, 2018; Wu et al., 2024). Specifically, controlled retrieval mechanisms appear to be largely preserved in healthy aging, though they operate with reduced neural efficiency (Martin et al., 2022). This maintained performance, particularly when processing more challenging or atypical concepts (Alves et al., 2021), is supported by a compensatory overactivation and functional reorganization of the brain’s underlying network architecture (Alves et al., 2023; Martin et al., 2023).

When considered together, the canonicity and relatedness x diagnosis interaction terms of the trial-level model, the *post-hoc* group comparisons across canonicity and relatedness conditions, and the group comparisons of the Canonicity and Relatedness scores, all suggest that both ADtyp and lvPPA showed greater impairment in controlled retrieval, but not selection, compared to controls. This pattern agrees with Corbett et al., 2015, who also reported significant canonicity and relatedness effects in the performance of this task in a group of AD patients; however, unlike the findings of Henderson et al. (2025b), we did not find markedly larger effects in the lvPPA than the ADtyp group in any of the analyses. A plausible explanation for this discrepancy is the composition of our ADtyp cohort: roughly half had an early-onset presentation ( < 65 years), a profile linked to more extensive cortical atrophy and executive-function deficits than late-onset cases (Tort-Merino et al., 2022; Joubert et al., 2016; Frisoni et al., 2007). Even though all individuals in the ADtyp cohort conformed to the typical memory-predominant presentation, several exhibited pronounced executive-function impairments, potentially inflating their sensitivity to the experimental Canonicity and Relatedness semantic-control manipulations.

### 4.2 Controlled retrieval and selection control mechanisms showed partially differentiable cognitive correlates

Although our results can only be considered exploratory due to the power limitations that come with small sample sizes, Canonicity (reflecting controlled retrieval) and Relatedness (reflecting semantic selection) scores exhibited partially distinct associations with other cognitive measures similar to findings in other recent work (Krieger-Redwood et al., 2025). The exploratory correlation analysis showed that, across patient groups, controlled retrieval, indexed by the Canonicity score, preferentially associated with measures of semantic knowledge, whereas semantic selection, indexed by the Relatedness score, preferentially associated with measures of executive function. The cognitive correlates analysis with linear models accounting for diagnosis, severity, and key demographic factors provides partially concordant results. The finding that two executive function measures, digit span backward and phonemic fluency, selectively predicted the Relatedness score but not the Canonicity score is consistent with the view that semantic selection draws more heavily on domain-general executive resources than controlled retrieval. However, the Canonicity score was not preferentially linked to semantic knowledge tests as animal verbal fluency predicted both Canonicity and Relatedness scores to a comparable. Finally, the intra-individual z-score analysis descriptively echoed this dissociation; patients were far more likely to show isolated impairment in one score than concurrent deficits in both. Though strictly exploratory, overall, our data suggest the existence of various cognitive mechanisms within the semantic cognition system that can be differentially impaired by neurodegeneration depending on the type and location of brain damage.

### 4.3 LvPPA performed the worse on all experimental task conditions

lvPPA scored consistently lower than both other groups on all conditions, though the difference with ADtyp did not reach statistical significance in any comparison. The trial-level model *post-hoc* comparisons showed lvPPA performed significantly worse in overall accuracy and in both non-canonical conditions. Various factors may explain the lvPPA group’s consistently worse performance. Most evidence from this study points toward a more robust and severe manifestation of controlled-retrieval deficits in lvPPA though other possibilities such as impairment of core semantic representations cannot be confirmed or disproved by this study. Severe controlled retrieval impairment could result in failure to provide stable access to representations even in low control demand conditions. This is supported by our condition-specific contrasts (Table 5), where the canonicity penalty was more pronounced in the lvPPA group (OR = 2.8, *p* = 0.004 vs. controls) than the ADtyp cohort (OR = 2.2, *p* = 0.047 vs. controls). Furthermore, a larger proportion of individuals in the lvPPA than the ADtyp group showed marked deficits in controlled retrieval demands, as indicated by a Canonicity score in the pathological range (Z-score > 1.65 vs. controls). Extensive GM and white matter damage in the posterior temporal parietal region, both known to be greater in atypical than typical AD (Caso et al., 2015; Madhavan et al., 2016; Phillips et al., 2024), could act as a putative underlying factor of lvPPAs worse performance. These findings provide initial evidence for distinct mechanisms contributing to semantic impairment in lvPPA that need to be further studied and refined in future work.

### 4.4 Increasing control demands across experimental task conditions revealed differential recruitment of the semantic-cognition brain network

Our exploratory neuroimaging analyses, while descriptive due to the modest sample size, revealed a graded pattern in which the least semantic-control-demanding condition engaged relatively anterior regions, while increasingly demanding conditions recruited additional frontal, posterior-lateral temporal, and parietal cortices. The canonical-unrelated condition, the least taxing for semantic control, correlated chiefly with two gray-matter clusters in the bilateral dorsolateral anterior temporal lobe. Under higher control demands, these correlations spread into broader fronto-temporo-parietal regions, and significant associations also emerged in the right hemisphere including the inferior temporal, inferior frontal, and middle frontal gyri. Although these unadjusted, TIV-corrected voxel-wise mappings must be interpreted with caution as tentative trends, this anterior-temporal focus accords with studies showing key involvement of this region in both verbal (Domoto-Reilly et al., 2012) and non-verbal (Libon et al., 2013) semantic tasks in AD, whereas the wider fronto-temporo-parietal pattern aligns with the typical semantic-control network described in recent lesion-mapping and meta-analytic work (Hodgson et al., 2024; Souter et al., 2022). The right-hemisphere involvement may reflect contributions of the non-verbal nature (Jackson, 2021) of our stimuli, possible recruitment of domain-general control circuitry (Hodgson et al., 2024), and /or disease severity.

### 4.5 Different semantic impairment mechanisms showed overlapping but distinct neural signatures

Previous evidence (Badre et al., 2005; Nagel et al., 2008) shows that retrieving less-salient knowledge, or controlled retrieval, and resolving semantic competition engage distinct neural circuits. Consistent with this view, our exploratory analysis observed that the Canonicity and Relatedness scores showed overlapping but distinct neuro-anatomical correlates that mirrored their cognitive profiles. The Canonicity score (reflecting controlled-retrieval) was linked to the widest set of regions, spanning areas implicated in both semantic cognition and domain-general control across both hemispheres. The Relatedness score (reflecting selection) was the least “posterior” of the scores, associated with only a few small clusters centered on the lateral MTG and bilateral inferior and middle frontal gyri. The left posterior ITG, fusiform gyrus, and posterior MTG clusters, core nodes of the semantic-control network (Jackson, 2021), likely facilitate retrieval of weak associations, while ATL and angular gyrus involvement would underpin more automatic semantic tasks (Jefferies et al., 2020). Bilateral MFG and IFG clusters point to domain-general control engagement (Hodgson et al., 2024; Jackson, 2021; Thompson et al., 2022), and medial-parietal involvement, part of the extended semantic network (Jung and Lambon Ralph, 2023), may reflect interplay with hippocampal memory circuitry (Binder and Desai, 2011). As expected, the left IFG, a region long tied to competitive selection (Thompson-Schill et al., 1997), was prominent in the relatedness map, and smaller right IFG and bilateral MFG clusters again implicate domain-general control, matching the executive-function links of that score.

The ROI analyses mirrored the whole-brain results in that it also showed overlapping and distinct neural correlates for the Canonicity and Relatedness scores. The results showed that GM volume in all three ROIs was a significant predictor of the Canonicity score whereas only pMTG volume significantly predicted the Relatedness score. Therefore, our initial hypotheses expecting controlled retrieval would be preferentially associated with atrophy in key areas of the semantic control network (IFG and pMTG), and selection with atrophy in frontal areas included in both the semantic and domain-general cognitive control systems (MFG), was not confirmed in our sample. Overall, these imaging results, while limited by the small sample size and exploratory nature, provide initial evidence that semantic impairments in AD can emerge through damage to multiple differentiable brain circuits.

## 5 Strengths and limitations

This study has several limitations that warrant discussion. First, the modest sample size significantly limited statistical power in various analyses, particularly for the voxel-based morphometry analyses aimed at detecting subtle neuro-anatomical correlates. Consequently, we placed special caution labeling these analyses as exploratory and discussing the implications of this limitation throughout the manuscript. We also tried to mitigate power concerns by using various types of analyses, including mixed-effects models that leverage trial-level data, to address the research questions and placing emphasis on the results that converged across different methodologies. Despite this, we recognize our results require replication in larger samples. This study’s AD cohort is CSF biomarker confirmed and among the largest to complete a task purpose-built to probe semantic-control processes, however atypical AD phenotypes, including lvPPA, are intrinsically rare, and multi-center collaborations will probably be needed to validate findings. Furthermore, we can also guarantee that none of the study participants presented significant cerebrovascular disease on neuroimaging as this is a basic exclusion criteria of the SPIN cohort. We acknowledge the inherent boundaries of *in vivo* diagnostic categorization. Co-pathologies, most notably TDP-43, are known to co-occur in both amnestic AD and lvPPA. Given the current lack of widely available *in vivo* biomarkers for these mixed pathologies, ruling them out completely is technically unfeasible at this time, representing a limitation inherent to current clinical research. Second, although the experimental paradigm was designed as a non-verbal semantic-judgment task, each trial included an orthographic and auditory cue describing the required action, thereby introducing a verbal component. During study planning, we recognized that this hybrid format might restrict conclusions about modality-specific semantic processing; however, its prior validation, together with the ability to manipulate controlled retrieval and selection within a single paradigm, outweighed that concern. Moving forward, we intend to supplement these data with purely verbal semantic-control tasks to disentangle modality effects more precisely.

## 6 Conclusion

In this study, we set out to elucidate the cognitive processes and neuroanatomical substrates of semantic control in AD. Using a previously validated non-verbal semantic-judgment task tailored to probe semantic control, we found that both patients and controls were affected by the increasing semantic control demands of the task. Both patient groups, the typical memory impaired presentation of AD and the atypical language dominant presentation (lvPPA), were impaired relative to controls in the controlled retrieval, but not the selection, semantic control mechanism. Furthermore, lvPPA scored consistently lower than the other groups in all conditions of the experimental task, even the condition with the lowest semantic control demands, suggesting greater presence of other types of semantic impairment. Correlations with neuropsychological performance and structural MRI revealed distinctive yet partly overlapping cognitive and neural signatures for each mechanism, suggesting different degrees of reliance on semantic-specific versus domain-general control networks. This is consistent with the view that neurodegenerative disease can differentially impact distinct aspects of semantic control depending on the topography of brain atrophy. These results refine current models of semantic dysfunction in AD and highlight cognitive and neural targets that could inform differential diagnosis, symptom management, and rehabilitation planning. Future research comparing verbal and non-verbal modalities and extending the framework to other neurodegenerative syndromes will further clarify modality effects and the generalizability of these mechanisms.

## Data Availability

The data analyzed in this study is subject to the following licenses/restrictions: The task stimuli material is available on the open research data repository: https://doi.org/10.34810/data2771. The de-identified datasets used and/or analyzed during the current study are available from the corresponding author on reasonable request. Requests to access these datasets should be directed to MS-S, MSantosS@santpau.cat.

## Footnotes

1
www.r-project.org
