# Supplementary material for: Semantic control across Alzheimer’s disease phenotypes: cognitive mechanisms and neural correlates
Source: Front Aging Neurosci. 2026 Aug 12;18:1821431. doi: 10.3389/fnagi.2026.1821431 (PMC13506867; doi:10.3389/fnagi.2026.1821431)
Supplement: Supplementary file 1 [file Data_Sheet_1.docx]

Supplementary Material

# 1 Outlier analysis

Trials were identified as outliers if:

- the overall accuracy for a specific item in the control group was below 50%: 8 trials: (i) paint a wall (noncanonical unrelated & noncanonical related conditions); (ii) dust off an ornament (noncanonical related condition); (iii) hammer a nail into wood (noncanonical related condition); (iv) put up a poster (canonical related & noncanonical related conditions); (v) kill a fly (noncanonical unrelated condition); (vi) and chop vegetables (noncanonical related condition); or
- the control group consistently selected an incorrect answer over the correct one: 2 trials: (i) paint a wall (noncanonical unrelated condition); and (ii) dust off an ornament (noncanonical related condition).

# 2 Supplementary Figures

**Supplementary Figure 1.** GM volume and accuracy. 0: Correlation of GM volume with total accuracy. A-G: Correlation of GM volume with accuracy under each of the 4 conditions for patients. Total intracranial volume was included as a covariate and maps were thresholded at *p* < .005 uncorrected at the voxel level with a cluster extent threshold of ~ **100 mm³**. The colour bar reflects t-values from the voxel-wise statistical analysis: A) canonical unrelated condition; B) canonical related condition; C) noncanonical unrelated condition; and D) noncanonical related condition; and with the scores: E) canonicity score; and F) relatedness score.

# 3 Supplementary Tables

**Supplementary Table 1.** Goodness-of-Fit and Information Criterion Metrics for Nested Generalized Linear Mixed Models (N = 28 patients, 3,136 total trials). The table contrasts the baseline model against the expanded model containing the third interaction term. Model comparison is evaluated using marginal and conditional coefficients of determination (R2), Akaike Information Criterion weights adjusted for small samples (AICc weights), and a Likelihood Ratio Test (χ2).

| **Model** | **R2 (cond.)** | **R2 (marg.)** | **AICc weights** | **χ2** | **p_**  **value** |
| --- | --- | --- | --- | --- | --- |
| correct response ~ Relatedness * DX + Canonicity * DX + Age + Gender + Education years + (1\|participant) + (1\|Task ID) + (1\|correct position) | 0.536 | 0.223 | 0.206 | - | - |
| correct response ~ Relatedness * DX + Canonicity * DX +Relatedness * Canonicity + DX + Age + Gender + Education years + (1\|participant) + (1\|Task ID) + (1\|correct position) | 0.531 | 0.232 | 0.794 | 4.581 | 0.019 |

**Supplementary Table 2.** MM-estimation robust regression: neuropsychological predictors of the canonicity and relatedness scores (patients only, N = 23–28). Each row represents a separate model. Covariates in all models: age, years of education, sex, diagnosis (ADtyp = 0, lvPPA = 1), and MMSE. All neuropsychological predictors were standardised within the patient sample (z-scored; higher = better; TMT-B negated before standardisation). β = standardised regression coefficient; 95% CI = 95% confidence interval.

|  | **Canonicity score** | | | **Relatedness score** | | |
| --- | --- | --- | --- | --- | --- | --- |
| **Neuropsychological test** | ***β*** | **95% CI** | ***p*** | ***β*** | **95% CI** | ***p*** |
| **Semantic knowledge** | | | | | | |
| Animal fluency | **-2.21^**^** | [-3.42, -0.99] | **.001** | **-1.52^**^** | [-2.40, -0.63] | **.002** |
| Synonyms | -0.28 | [-3.58, +3.02] | .860 | -0.39 | [-2.31, +1.54] | .680 |
| BNT (spontaneous) | +0.22 | [-1.64, +2.07] | .808 | -0.12 | [-1.77, +1.54] | .883 |
| **Executive function** | | | | | | |
| Digit span backward | -0.67 | [-2.59, +1.26] | .476 | **-1.70^*^** | [-2.93, -0.46] | **.010** |
| TMT-B | -0.40 | [-2.22, +1.42] | .648 | -0.76 | [-2.02, +0.50] | .221 |
| Phonemic fluency (p) | -1.56 | [-4.57, +1.44] | .288 | **-1.24^*^** | [-2.47, -0.02] | **.047** |

******* *p < .05;* ******** *p < .01; † p < .10 (trend). Bold values = p < .05. All results uncorrected for multiple comparisons; findings should be interpreted as exploratory. Abbreviations: BNT = Boston Naming Test; TMT-B = Trail Making Test Part B (negated: higher = better); p = phonemic fluency (words beginning with letter p, 1 min).*
